# Species distribution and antifungal drug susceptibilities of yeasts isolated from the blood samples of patients with candidemia

**DOI:** 10.1038/s41598-019-40280-8

**Published:** 2019-03-07

**Authors:** Erika Lindberg, Helena Hammarström, Nasser Ataollahy, Nahid Kondori

**Affiliations:** 1 Department of Infectious Diseases, The Sahlgrenska Academy, University of Gothenburg, Gothenburg, Sweden; 2 Department of Clinical Microbiology, Sahlgrenska University Hospital, Gothenburg, Sweden

## Abstract

*Candida albicans* is the most frequently isolated fungal species in hospital settings worldwide. However, non-albicans *Candida* species with decreased susceptibility to antifungals have emerged as an important cause of fungemia. The aims of this study were to determine the species distribution of fungi isolated from the blood samples of patients at a Swedish University Hospital and to define the *in vitro* susceptibilities of these isolates to nine antifungal agents. In total, 233 yeast isolates from 143 patients were included in this study. Antifungal susceptibility testing was performed using broth dilution Sensititre YeastOne panels, which comprised amphotericin B, 5-flucytosine, fluconazole, itraconazole, voriconazole, posaconazole, anidulafungin, micafungin, and caspofungin. The most common species in all age groups was *C*. *albicans* (n = 93, 65%), followed by *C*. *glabrata* (n = 27, 19%) and *C*. *parapsilosis* (n = 15, 10%). *C*. *glabrata* was mostly found in elderly individuals, while *C*. *parapsilosis* was found mainly in young children (p = 0.008). Antifungal resistance was low in the *Candida* species, except for reduced susceptibility to fluconazole among *C*. *glabrata* strains. *C*. *albicans* is the most frequent colonizer of Swedish patients. In general antifungal resistance is uncommon in *Candida* species. Nevertheless, reduced susceptibilities to fluconazole and echinocandins were found in *C*. *glabrata* and *C*. *parapsilosis*, respectively.

## Introduction

The increased application of antifungal agents for prophylactic or empirical treatment has led to a change in the epidemiology of fungemia and the emergence of fungal pathogens with decreased susceptibility or resistance to antifungal drugs^1^. While *Candida albicans* is the most frequently isolated fungal species in the hospital setting worldwide, non-albicans *Candida* species with decreased susceptibility to antifungals have emerged as an important cause of fungemia^1^. The treatment of fungal infections is increasingly problematic owing to increased resistance to antifungal agents among *Candida* species^2^. Antifungal susceptibility patterns vary among *Candida* species and may influence the clinical outcomes for infected patients^3^.

*Candida glabrata* has intrinsically lower susceptibility to fluconazole, and may develop cross-resistance to other azoles. Furthermore, the frequency of resistance to echinocandins is increasing among *Candida* species^4^. Therefore, antifungal susceptibility testing is crucial for the management of patients with invasive *Candida* infection^5^. There are two internationally recognized standard methods for antifungal susceptibility testing using minimum inhibitory concentration (MIC), as developed by the European Committee on Antimicrobial Susceptibility Testing (EUCAST) and the Clinical and Laboratory Standards Institute (CLSI)^6,7^. However, these methods are time-consuming and are not practical tools for antifungal susceptibility testing in clinical laboratory use. This has led to the development of commercially available tests, such as the Etest (bioMerieux, Marcy-l'Étoile, France), VITEK (bioMerieux), and Sensititre YeastOne (SYO; Thermo Fisher Scientific, MA, USA) systems, as alternatives to the standard broth microdilution methods. The SYO method represents a simple, flexible, easy-to-handle and time-saving alternative for antifungal susceptibility testing for daily use in the routine clinical laboratory. It has been used widely with excellent results in terms of accuracy and reproducibility^8–11^.

The aims of this study were to determine the species distribution and the antifungal susceptibility patterns of fungi isolated from blood samples collected from patients with suspected septicemia’, over a period of 3.5 years at a Swedish University Hospital. *In vitro* antifungal susceptibility testing of fungi isolated from blood was conducted using nine antifungal agents in the SYO panel, i.e., amphotericin B, 5-flucytosine, fluconazole, itraconazole, voriconazole, posaconazole, anidulafungin, micafungin, and caspofungin.

## Results

### Species distribution

*Candida* species were recovered from 0.1% of all the blood cultures collected from patients with suspected septicemia. Overall, 233 isolates were collected from 143 patients (84 males and 59 females) during the period of January 2013 to June 2016. The mean and median ages were 56 and 63 years, respectively, with an age range of 3–96 years.

#### The fungal species distribution was as follows

*C*. *albicans*, 93 (65%); *C*. *glabrata*, 27 (19%); *C*. *parapsilosis*, 15 (10%); *C*. *dubliniensis*, 6 (4%); *C*. *tropicalis*, 4 (3%); *C*. *krusei*, 3 (2%); and others (*C*. *kefyr*, *C*. *lusitaniae*, *C*. *sake* and *C*. *pelliculosa*), 4 (3%) (Table 1). One isolate of *Saccharomyces cerevisiae* was identified. From 98/143 patients, only one fungal isolate was recovered, while two or more (up to seven) isolates were recovered from the remainder of the patients (45/143) (mean, 1. 6).

**Table 1:** Fungal species distribution among patients with positive blood cultures.

Fungal species	Patients N (%)	Isolates n (%)	Gender		Age Mean ± SD
			Male (N)	Female (N)	
*Candida albicans*	93 (65)	142 (61)	53	40	58 ± 23
*Candida glabrata*	27 (19)	36 (15)	17	10	63 ± 24
*Candida parapsilosis*	15 (10)	29 (12)	10	5	31 ± 27
*Candida tropicalis*	4 (3)	5 (0.2)	2	2	60 ± 8
*Candida krusei* (*Pichia kudriavzevii*)	3 (2)	6 (2.5)	2	1	53 ± 10
*Candida dubliniensis*	6 (4)	6 (2.5)	3	3	55 ± 26
*Other Candida species*	4 (3)	8 (3)	2	2	62 ± 9
*Saccharomyces cerevisiae*	1 (0.7)	1 (0.4)		1	70
Total	143	233	84	59	56 ± 25

**Figure 1:**
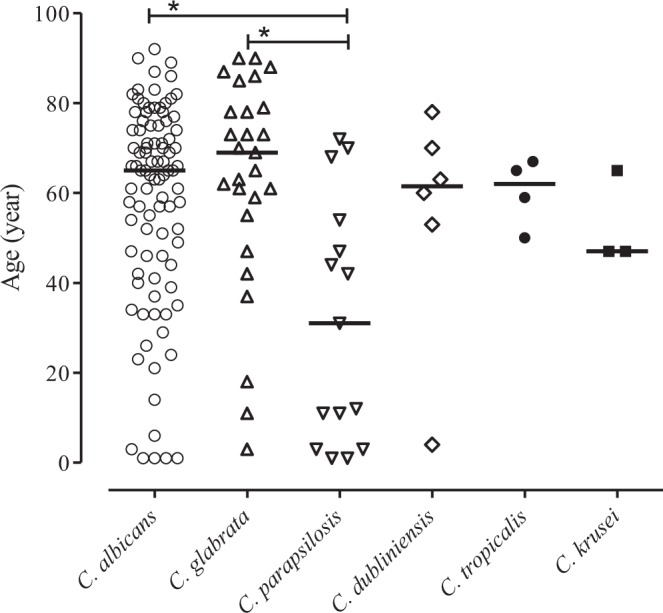
*Candida* species isolated from the blood samples of patients with candidemia, classified according to age (years), **p < 0.05; ***p < 0.005.

Eight of the patients had more than one *Candida* species. Four patients were coinfected with *C*. *albicans* and *C*. *glabrata*; one patient with *C*. *albicans* and *C*. *lusitaniae*; and one patient had both *C*. *albicans* and *C*. *tropicalis*. Two patients had three different *Candida* species: one had *C*. *albicans*, *C*. *glabrata* and *C*. *krusei*, while the other had *C*. *albicans*, *C*. *glabrata* and *C*. *dubliniensis*. Figure 1 shows the species distribution and the ages of the patients. *C*. *glabrata* was significantly more common among elderly patients, while *C*. *parapsilosis* was significantly more frequently isolated from younger patients (p < 0.05). *C*. *albicans* was detected in all age groups.

**Figure 2:**
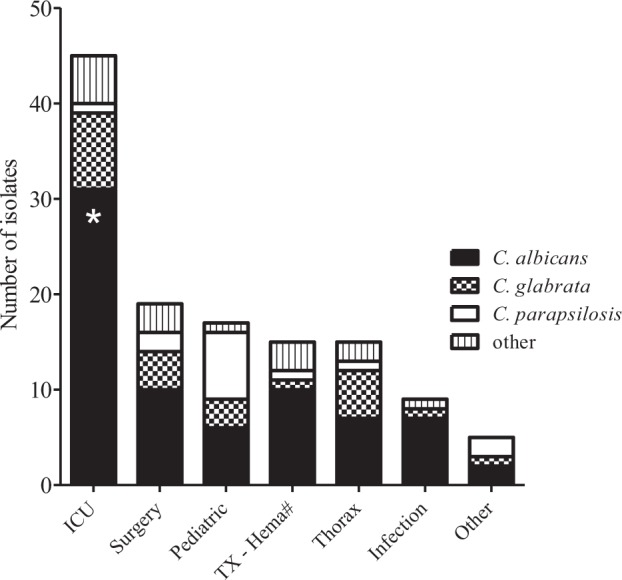
Numbers of *Candida* species isolated from patients with candidemia who were hospitalized in different hospital units. *Candida albicans was found significantly more often in ICU wards (p < 0.05). ^#^Transplantation and Hematology unit.

The blood cultures were collected from patients who were admitted at different clinical units. The majority of the samples were collected from Sahlgrenska University Hospital, while some samples were collected from patients who were hospitalized at regional hospitals. Overall, there were 45 patients (31%) at ICUs, 19 patients (13%) at surgical wards, 17 patients (12%) at pediatric units, 15 patients (10%) at hematology and transplantation units, 15 patients (10%) at the thoracic medicine department, and 9 patients (6%) at the infectious disease unit. Figure 2 shows the species distribution in relation to the unit to which the patient was admitted. Candidemia caused by *C*. *albicans* was found in patients admitted to all the types of wards/units. However, *C*. *albicans* was significantly more common in the patients in the ICU than in other clinical units (p < 0.05). *C*. *glabrata* was commonly found in the ICU, thoracic and surgery wards, while *C*. *parapsilosis* was the most commonly found species among younger patients in the pediatric units. However, these did not reach significance level.

### Antifungal susceptibility patterns

The MIC values obtained for nine antifungal agents among the *C*. *albicans*, *C*. *glabrata* and *C*. *parapsilosis* isolated from the blood samples are summarized in Table 2. When applying the EUCAST and CLSI clinical breakpoints (CBPs) we found that all *C*. *parapsilosis* isolates and all except one isolate of *C*. *albicans* were susceptible to fluconazole. Overall, 97% of all *C*. *glabrata* isolates showed reduced susceptibility to fluconazole (MIC_90_ = 16 µg/ml). The MIC values for posaconazole were overall low, and only one isolate of *C*. *albicans* and one isolate of *C*. *parapsilosis* were found to be resistant when applying the EUCAST CBPs. Only one isolate of *C*. *albicans* was found to be resistant to voriconazole. This isolate was also resistant to other tested azoles (posaconazole and fluconazole). Applying the EUCAST CBPs, anidulafungin was revealed to be the antifungal drug to which the *Candida* isolates showed reduced susceptibility. Twenty-four *C*. *albicans* (17%) and two *C*. *glabrata* isolates, found in 20 patients, were not susceptible to anidulafungin. However, when the CLSI CBPs were applied, all the isolates exhibited susceptibility to anidulafungin. Applying the epidemiological cutoff values (ECVs), almost all the isolates had wild-type phenotype drug susceptibility to echinocandins, amphotericin B, and5-flucytosine. Only one *C*. *glabrata* isolate exhibited a non-wild-type phenotype with respect to susceptibility to 5-flucytosine.

**Table 2:** Minimal inhibitory concentrations (MIC_50_ and MIC_90_) of amphotericin B, 5-flucytosine, fluconazole, itraconazole, voriconazole, posaconazole, anidulafungin, micafungin, and caspofungin for *Candida* species isolated from the blood samples of patients with candidemia.

Candida species	Antifungal agent	MIC (µg/ml)			Susceptible isolates (%)		ECV^&^	Isolates (%)	
		Range	MIC_50_	MIC_90_	EUCAST	CLSI		wt	non-wt
*C. albicans* (n = 142)	Amphotericin	0.25–1	0.5	1	100	ND	>2	100	0
	5-Flucytosine	0.06–0.5	0.06	0.12	ND	ND	>0.5	100	0
	Fluconazole	0.12–4	0.25	0.5	99	99			
	Itraconazole	0.015–0.12	0.03	0.06	97	100			
	Voriconazole	0.008–0.25	0.008	0.015	99	99			
	Posaconazole	0.008–0.12	0.015	0.03	99	ND*			
	Anidulafungin	0.015–0.12	0.03	0.06	83	100	>0.25	100	0
	Micafungin	0.008–0.06	0.008	0.015	97	100	>0.06	100	0
	Caspofungin	0.015–0.12	0.03	0.06	ND	100	>0.25	100	0
*C. glabrata* (n = 35)	Amphotericin	0.25–2	1	1	97	ND	>2	100	0
	5-Flucytosine	0.06–2	0.06	0.06	ND	ND	>0.25	99	1
	Fluconazole	2–128	16	16	97 (I**)	97 (SDD^†^)			
	Itraconazole	0.25–1	0.5	1	ND	3			
	Voriconazole	0.06–2	0.25	1	ND	ND			
	Posaconazole	0.12–2	1	2	ND	ND			
	Anidulafungin	0.015–0.06	0.03	0.03	94	100	>0.125	100	0
	Micafungin	0.008–0.03	0.015	0.015	100	100	>0.06	100	0
	Caspofungin	0.03–0.25	0.06	0.12	ND	100	>0.25	100	0
*C. parapsilosis* (n = 29)	Amphotericin	0.12–1	0.25	0.5	100	ND	>1	100	0
	5-Flucytosine	0.06–0.25	0.06	0.06	ND	ND	>0.5	100	0
	Fluconazole	0.25–2	0.5	2	100	100			
	Itraconazole	0.015–0.25	0.03	0.12	97	97			
	Voriconazole	0.008–0.06	0.015	0.03	100	100			
	Posaconazole	0.015–0.12	0.03	0.06	97	ND			
	Anidulafungin	0.25–2	0.5	2	100 (I)	100	>8	100	0
	Micafungin	0.5–2	1	2	100 (I)	100	>4	100	0
	Caspofungin	0.12–1	0.25	0.5	ND	100	>2	100	0

^&^Epidemiological cutoff value. *Not determined. **Intermediate category. ^†^Susceptible dose-dependent.

## Discussion

Here, we report that three *Candida* species accounted for more than 90% of cases of candidemia (*C*. *albicans*, *C*. *glabrata* and *C*. *parapsilosis*) in the western part of Sweden during the period 2013–2016. *C*. *albicans* was the most common cause of candidemia, followed by *C*. *glabrata* and *C*. *parapsilosis*. This is in agreement with previous studies that reported *C*. *albicans* as the most commonly isolated fungus from blood samples^9^. Compared with a study from 1987 conducted in the same geographic area of Sweden, the frequency of *C*. *albicans* candidemia in the present study is reduced from 70% to 65%^12^. Historically, *C*. *albicans* has been recognized as the most frequently identified yeast in blood cultures^13,14^. However, more recent studies have shown a decreasing frequency of *C albicans* candidemia, while the frequencies of *C*. *glabrata* and *C*. *krusei* candidemia have remained stable and those of *C*. *parapsilosis* and *C*. *tropicalis* are increasing^14^. These changes in patterns of detection may reflect the use of more advanced and standardized methods, such as MALDI-TOF, which have led to more accurate identification of yeast species, as compared to conventional methods. The reported distributions of *Candida* species in blood samples vary across studies conducted in different geographic areas^14^. In Northern Europe and the USA, *C*. *albicans* is still the most common fungal species found in blood samples, whereas studies from Brazil, Iran, and Spain report non-albicans *Candida* as the most frequent cause of candidemia^14,15^. *C*. *parapsilosis* was found to be the major cause of candidemia in Iran^15^. In the present study, *C*. *parapsilosis* candidemia was associated with younger age, as compared to candidemia caused by *C*. *glabrata* and *C*. *albicans*. This finding is in line with previous studies that have reported *C*. *parapsilosis* as the most prevalent *Candida* species among children and neonates^14,16^. This observation remains unexplained. However, the prevalence of *C*. *parapsilosis* in children may reflect the use of intravascular devices to treat neonates^17^.

It has been suggested the infection with *C*. *glabrata* is more common in elderly patients^15,18^. This association may be attributable to earlier treatment with antifungal drugs or the nature of the underlying disease. Lockhart *et al*. reported increased *C*. *glabrata* colonization in the oral cavities of elderly patients^19^. Our present study also supports the notion that *C*. *glabrata* is more common among elderly patients. The mean age of the patients infected with *C*. *glabrata* in our study was 63 ± 24 years, which is comparable to the results from other studies^18,20^.

In the present study, antifungal susceptibility was determined using the commercially available SYO method. Antifungal resistance was found only in *C*. *glabrata*, where 97% showed decreased susceptibility to fluconazole. Since no CBPs have yet been established specifically for commercial antifungal susceptibility testing, such as SYO, the MIC values obtained by the SYO method must be interpreted using the EUCAST and CLSI CBPs^8^. When applying the EUCAST CBPs, we found that all of the *C*. *parapsilosis* (29/29) isolates and 94% of the *C*. *glabrata* (33/35) isolates were classified as having intermediate susceptibility to anidulafungin. However, according to the CLSI CBPs, these isolates were categorized as susceptible to anidulafungin. To detect resistant isolates, some investigators have recommended the use of CLSI interpretive criteria for the interpretation of MIC results instead of EUCAST^21^. Further studies are needed to establish species-specific CBPs for susceptibility testing by SYO.

Overall, *C*. *albicans* was the most commonly isolated species from the blood samples of patients with candidemia. *C*. *glabrata* was more common among elderly patients and *C*. *parapsilosis* was more frequently isolated from children and younger patients. Reduced susceptibility to antifungal drugs was rarely seen in *Candida* species isolated from blood. However, the SYO method needs to be refined in terms of resolving the discrepancies noted in the susceptibility patterns defined using the EUCAST and CLSI CBPs.

## Methods

### Fungal Isolates

In total, 153,712 blood culture bottles (BactAlert; bioMerieux, Marcy-l'Étoile, France) with samples from 51,269 patients were cultured in the period from January 2013 to June 2016 at the Department of Clinical Microbiology, Sahlgrenska University Hospital, Gothenburg, Sweden. A total of 233 (0.002%) positive yeast isolates from 143 (0.003%) patients was collected during this period. Yeast-positive blood cultures were inoculated on Sabouraud agar and CHROMagar Candida (Becton Dickinson, Franklin Lakes, NJ, USA) plates and incubated overnight at 37 °C. The yeast isolates were identified to the species level using matrix-assisted laser desorption/ionization-time of flight (MALDI-TOF) (VITEK-MS; bioMerieux), together with macroscopic and microscopic observations of cell morphology. In addition, green colonies on the CHROMagar *Candida* plates were tested with a commercial kit (BICHRO-DUBLI FUMOUZE; Fumouze Diagnostics, Levallois Perret, France) to distinguish *Candida dubliniensis* from *C*. *albicans*, according to the manufacturer’s instructions. This method is based on the agglutination of blastopores of *C*. *dubliniensis* with latex particles coated with a monoclonal antibody that is specific for a *C*. *dubliniensis* surface antigen.

### Antifungal susceptibility testing

Sensititre YeastOne panels (Trek Diagnostic Systems, Thermo Scientific, East Grinstead, West Sussex, UK) were used for antifungal susceptibility testing. The plates contained serial twofold dilutions of amphotericin B (0.12 to 8 mg/L), 5-flucytosine (0.06 to 64 mg/L), fluconazole (0.12 to 256 mg/L), itraconazole (0.015 to 16 mg/L), voriconazole (0.008 to 8 mg/L), posaconazole (0.008 to 8 mg/L), anidulafungin (0.015 to 8 mg/L), micafungin (0.008 to 8 mg/L), and caspofungin (0.008 to 8 mg/L).

Antifungal susceptibility testing was performed by SYO according to the instructions provided by the manufacturer. *Candida parapsilosis* ATCC 22019 from the American Type Culture Collection (ATCC 22019) and *Candida krusei* ATCC 6258 were included as control strains in all the experiments. Minimum inhibitory concentrations (MICs) were determined after 24 h of incubation at 34–35 °C. The MIC was defined as the lowest concentration of antifungal agent at which the color in the well changed from red (positive, indicating growth) to blue (negative, indicating no growth).

### Interpretation of MIC results

Interpretation of susceptibility was performed by applying the CBPs defined by EUCAST and CLSI^6,22^. In the absence of CBPs, isolates were defined as having a wild-type or a non-wild-type drug susceptibility phenotype (to amphotericin, 5-flucytosine, anidulafungin, micafungin, and caspofungin) according to the epidemiological cutoff values (ECV), as shown in Table 2^23^.

### Statistical analysis

The data were analyzed using the Kruskal-Wallis test to avoid random significance when comparing several groups. Significance was set at a P-value of <0.05 (two-tailed). All analyses were done using the GraphPad Prism ver. 4.00 software (GraphPad Inc., San Diego, CA, USA).

### Ethical statement

Ethical approval and patient consensus was not considered necessary due to the descriptive nature of the study that implied only the samples obtained during routine laboratory activity.

## Data Availability

All isolates and the data that support the findings of this study are available from the corresponding author upon request.
